# Brain function assessment in different conscious states

**DOI:** 10.1186/1753-4631-4-S1-S6

**Published:** 2010-06-03

**Authors:** Murat Ozgoren, Onur Bayazit, Sibel Kocaaslan, Necati Gokmen, Adile Oniz

**Affiliations:** 1 Department of Biophysics, Faculty of Medicine, Dokuz Eylul University, Izmir, 35340, Turkey; 2 Department. of Anesthesiology, Faculty of Medicine, Dokuz Eylul University, Izmir,35340, Turkey

## Abstract

**Background:**

The study of brain functioning is a major challenge in neuroscience fields as human brain has a dynamic and ever changing information processing. Case is worsened with conditions where brain undergoes major changes in so-called different conscious states. Even though the exact definition of consciousness is a hard one, there are certain conditions where the descriptions have reached a consensus. The sleep and the anesthesia are different conditions which are separable from each other and also from wakefulness. The aim of our group has been to tackle the issue of brain functioning with setting up similar research conditions for these three conscious states.

**Methods:**

In order to achieve this goal we have designed an auditory stimulation battery with changing conditions to be recorded during a 40 channel EEG polygraph (Nuamps) session. The stimuli (modified mismatch, auditory evoked etc.) have been administered both in the operation room and the sleep lab via Embedded Interactive Stimulus Unit which was developed in our lab. The overall study has provided some results for three domains of consciousness. In order to be able to monitor the changes we have incorporated Bispectral Index Monitoring to both sleep and anesthesia conditions.

**Results:**

The first stage results have provided a basic understanding in these altered states such that auditory stimuli have been successfully processed in both light and deep sleep stages. The anesthesia provides a sudden change in brain responsiveness; therefore a dosage dependent anesthetic administration has proved to be useful. The auditory processing was exemplified targeting N1 wave, with a thorough analysis from spectrogram to sLORETA. The frequency components were observed to be shifting throughout the stages. The propofol administration and the deeper sleep stages both resulted in the decreasing of N1 component. The sLORETA revealed similar activity at BA7 in sleep (BIS 70) and target propofol concentration of 1.2 µg/mL.

**Conclusions:**

The current study utilized similar stimulation and recording system and incorporated BIS dependent values to validate a common approach to sleep and anesthesia. Accordingly the brain has a complex behavior pattern, dynamically changing its responsiveness in accordance with stimulations and states.

## Background

**Figure 1:**
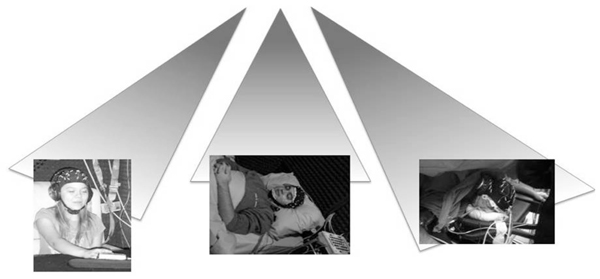
**The electrophysiological recordings** of a wake subject (left); recording during sleep (middle) and anaesthesia (right) with a similar EEG cap and stimulation system (EMISU).

The human brain represents one of the most complex biological systems in the world. When it comes to its computational power, the estimates are overwhelming and are not comparable to man-made systems [1]. This complex organ also has a very dynamic nature, during which it shifts from one state to another, almost entirely changing its functional properties. These major states can be exemplified in the case of conscious states. Hence the consciousness can also be defined with this concept of altered state of brain cognitive functioning. A perfect definition of consciousness is still not available; however the existence of differentiations between some conscious states is out of debate. The sleep, anesthesia and wakefulness are three of such separable states with distinct features (Figure 1).

The changes in the dynamic features of the brain can be obtained through its responsiveness. The conscious states are usually determined with the degree of lack of responses to outer world. Therefore a gradually changing response monitoring system can be useful in this type of approach. The auditory domain could be a proper stimulation modality, as it has been a common choice in neuroscience [2,3]. The auditory evoked potentials have been described with the acoustic features and stimulation patterns. Out of these sub features, the frequency, intensity and location are among the most well known properties [2,4]. Typically, tones (sinusoidal) or clicks are applied as the stimuli and the sensory/cognitive responses are recorded via electrodes attached to the scalp. The basic sensory features (bottom-up) are inherently accompanied with other features such as attention, memory (top-down) etc. Thus, the top-down and bottom-up properties all paint a complex landscape with very short latencies such as Auditory Brain-stem Responses (ABR), Middle Latency Responses (MLRs) to long latency wave forms [5]. A recently revisited auditory stimulation pattern has been the dichotic one, with a certain scope on brain asymmetry (syllables, tones etc.) [6-8]. From tones and clicks to syllables, these simple and semi-complex stimulations form a plausible domain for assessment of brain responsiveness. The need to obtain different features has forced the experimental design to include acoustical properties, attention-free assessment with mismatch, attention-bound oddball, dichotic and diotic features. Therefore these different properties have become a bundle of blocks constituting Brain biophysics battery (BBB).

On the other hand, the conditions of performing ideal experiments may not be an easy task as the operation room and the sleep environment are far from perfect neuropsychological setups. One of the major problems in surgery room comes from being electromagnetically hostile environment [9,10], therefore effecting the outcome of electrophysiological recording. During sleep, the ever changing body position as well as depth of sleep becomes problematic in conducting sensory/cognitive auditory tests (i.e. presenting perfect symmetrical and ideal auditory stimulations). Thirdly the stimulations especially in sleep may cause some alterations in the state of responsiveness of the brain [11].

Recently, the state of consciousness and brain functioning during sleep and anesthesia have become a topic of interest [5,12-16]. Furthermore Tung and Medelson [17] reviewed the studies and issues related to both sleep and anesthesia addressing components from neuromodulators to nuclei. Having noted above mentioned points, the present paper will address issues related to brain responsiveness in anesthesiology and sleep. The presentation of auditory stimulations with different physical and design related properties would enable the acquisition of some of the dynamic and complex response patterns of the brain.

## Methods

### Sleep procedure

The sleep experiments were performed in the Sleep Dynamics Research Laboratory of the Biophysics Department. The participants slept for one night in the laboratory and therefore, the current study is based on their first night sleep recordings. Overnight sleep data were collected from 12 healthy volunteer individuals (mean age: 24.5, range: 18-32, 10 males). Except one, all participants were right handed. The exclusion criteria included a past and/or present history of any neurological, psychiatric or chronic disorders and use of any drugs that effect cognitive functions. The participants were advised not to take any caffeine on the night of the recording day.

Participants arrived at the laboratory approximately two hours before their normal sleeping time and recordings were realized approximately between 23.00-08.00 o’clock. Over the entire course of the recording session, researchers stayed in the laboratory in which the recording systems were present. The participants spent a whole night lying on the bed in the isolated room which limited electromagnetic interference. Furthermore, the walls of the laboratory have been enveloped with an acoustic material to isolate room from the external auditory noise. The sleeping room was dimly lighted and the subject was monitored.

Bispectral index (BIS) recording was performed using a bispectral index monitor (Aspect-A2000) with a sensor (BIS Quatro). Sensors were checked for signal quality (SQI), assuring impedance below 5 kOhms. In every five seconds BIS was recorded via the RS232 cable using a HyperTerminal protocol. The BIS SQI values had been planned to be rejected below 50. However obtained SQI values were over 50; thus no rejection was required. Sleep scoring was performed according to the Rechtschaffen and Kales (R&K) [18] criteria in 30-s time windows. For every R&K score there were six BIS values (a BIS value obtained in every five seconds). Sleep data were analyzed in five BIS clusters, which have BIS values 90, 80, 70, 60, 50 respectively. For each stimulus, sweeps with 500 ms prestim and 1500 ms poststim data segments were formed in these five BIS clusters. The sweeps which had amplitudes higher than ±100 µV in Electrooculography (EOG) channel were automatically rejected. The recordings were corrected based on horizontal axis (baseline corrected) and digitally filtered with the 0.5-48 Hz band pass filter (12 dB/oct and zero phase shift, Neuroscan 4.3). Henceforth, every single subject’s data were averaged and all of the subjects’ grand average was prepared.

### Anesthesia procedure

The anesthesia related experiments were conducted in the surgery room. 12 subjects (9 females) participated with an age range of 25-46 (mean 37.75) years. These were patients with a similar surgery protocol undergoing for lumbar disc hernia. The ethical approval has been received from the local Ethics Committee. All patients have signed a consent form prior to procedures.

The propofol administration was controlled with a target-controlled infusion mechanism (Fresenius Vial Orchestra Base Primea, Le Grand Chemin, France) where precalculated brain dosage (effect-site) would be obtained. Accordingly, the concentration levels of 0 µg/mL to 1.6 µg/mL of Propofol were obtained. Besides the various monitoring devices, the bispectral index monitor was used. The BIS provides a dimensionless number from 0 to 100, which denotes to anesthesia depth. It utilizes electrophysiological (EEG) parameters including bispectral index. The wakefulness values range around >95 and with the propofol administration sudden drops in the index values occur. At around 1.0 µg/mL of Propofol administration, the activity would be in the range around 80. Commonly levels would be kept around 50-60 range to perform adequate surgery.

### Brain biophysics battery

The brain biophysics battery is an amalgamation of auditory blocks with different features:

Auditory evoked potential (AEP): 1500 Hz, 80 dBSPL, 500 ms, 2–3s inter stimulus interval (ISI). Unless mentioned otherwise throughout this manuscript AEP was used as the main example of stimulus condition.

Auditory event related potential (AERP): Auditory oddball paradigm applied via headphones (binaural). Auditory stimuli were 500 ms, 80 dBSPL, sinusoidal (target: 1600 Hz, nontarget:1500 Hz) sounds. Targets occurred in 20-25% of the cases.

Dichotic linguistic (DL): 36 different combinations of /ba/, /da/, /ga/, /ka/, /pa/, /ta/ syllables were represented by headphones, one from each ear. ISI was varied randomly between 2.5 and 3 seconds.

Dichotic tone (DT): Two different tones applied via headphones binaurally. Tones were 291 Hz and 392 Hz.

Mismatch negativity (MMN): A modified version of optimized MMN paradigm developed by Pakarinen et al. [19] was used. Standard tone was 75 ms in duration, at an intensity of 70 dB, composed of three sinusoidal partials (523 Hz as fundamental frequency) and presented simultaneously via headphones (resulting in perception of the sound source as localized in the centre). There were four type of deviants (duration, intensity, frequency, location), each with three levels. Location deviance was achieved by delaying the tone monaurally, right or left with equal probability.

These abovementioned stimuli were given in varying blocks from the onset to the end of the experimental sessions.

### EEG recording

A special stimulus unit developed in our lab Embedded Microcontroller Interactive Stimulus Unit (EMISU) [20] was used for the generation of stimuli and the recording of electrophysiological data both in sleep and anesthesia experiments. The EEG recording was achieved in the operation room by means of 40 channel EEG (Nuamps), headphones with noise cancellation feature (Creative HN-700, Republic of Singapore). MATLAB, EEGLAB, and EMISU software were used. The auditory stimuli were administered through noise cancelling head phones at a level of 85 dB SPL. The stimuli were consisted of tones 1500 Hz, 500 ms duration 30 ms rise/fall time, with randomly varying intervals ranging from 2.5 to 5 sec.

### EEG segment selection and data preparation

The continuous EEG data files (*.cnt) were epoched for the evoked condition or segmented for the spontaneous condition to equal data lengths. Each segment contained 601 data points (sampling rate 1000 Hz) which equaled to -200 to 400 ms for the epoched sections. Spontaneous segment would be selected from an artifact free section with no stimulation. In a section with stimulations, then the segment would be temporally separated by 2-3 seconds pre and post stimuli.

By means of manual observation the artifacts were removed. These artifact free sections were baseline corrected. The obtained sweeps were than averaged. Due to hostile conditions in the surgery room not all the channels were artifact free. For this reason 18 channels [21,22] with equal distribution across anterior/posterior and hemispheric locations were fixed across all subjects. These data segments were then transferred into standardized low resolution electromagnetic tomography (sLORETA) protocol. The data were transformed with proper electrode locations to obtain X, Y, Z coordinates, which were then used to create transformation matrix file in sLORETA application. “Global field power” (GFP) was used to estimate the target peaks. Therefore at a given sLORETA image the current density value (CDV) of maximal GFP peaks are presented.

### sLORETA procedures

sLORETA was applied to estimate the neural sources of event related scalp potentials [23,24] in the LORETA 2D potential distribution to 3D brain volume. Montreal Neurological Institute (MNI) was referred for standard solution space which was subdivided into a 3D grid consisting of 2394 volume elements with a regular cube size of 7x7x7 mm. An equivalent current dipole is positioned, for which a current density (µA/mm^2^) is computed on each of these voxels [23,24].

### Spectral representations

The spectrograms for the presentations of EEG data segments were obtained by means of a MATLAB routine [25]. The outline of the method is given below:

Wavelet transform of a signal  gives the time-scale information found by the Wavelet transform of a signal ***s***(***t***) gives the time-scale information found by the following formula

where the mother wavelet function  was chosen as Complex Morlet.

* denotes the complex conjugate, b is the shift parameter, the corresponding scale is given by dilation parameter a. Since most of the signal processing applications focus on the time-frequency analysis, it is essential to convert the scale information into frequency. The formula given below provides corresponding frequency ***F_a_*** for a specified scale value a

with the parameters ***F_c_*** denoting center frequency of the wavelet in Hz, Δ denoting the sampling period. For obtaining the spectrogram the Central (Cz) electrode data were used.

### Statistics

The Shapiro-Wilk test was used for determining distribution of the groups and deciding statistical methods. When the groups were in normal distribution paired t-test was used to compare the above mentioned two conditions. The correlation of BIS and propofol dosage was determined with nominal logistic regression.

## Results

The electrophysiological recordings, the auditory stimulations, and the BIS monitoring have been successfully performed in all subjects of the two experimental groups. Additionally in anesthesia group, drug infusion system was used. The conditions for the stimulations were kept similar.

### The anesthesia experiment

All subjects have undergone the step-wise propofol administration. One subject’s data were not available for both BIS and perfusion control system, therefore it was dismissed. The subjects had been instructed to stay awake. Around 0.8-1.2 µg/mL (propofol) they have lost consciousness (LOC) at which point they ceased to respond to comments. The electrophysiological recordings were continued regardless of LOC till reaching level of 1.6 µg/mL. Therefore all subjects received a similar dosage regime of propofol as well as auditory stimulations.

**Figure 2:**
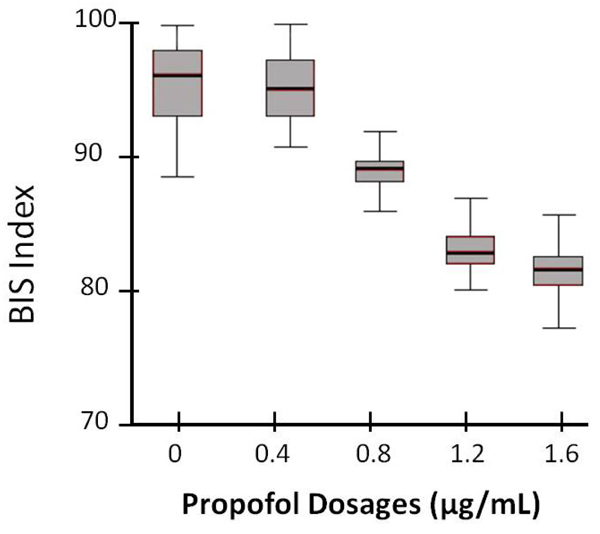
**BIS and propofol administration box plot.** The vertical axis denotes the BIS index values, whereas the horizontal axis present the propofol dosage (target effector site concentration, in µg/mL). The boxes represent the distribution of BIS versus the propofol. The outliers show the standard error.

The figure 2 represents the distribution of BIS values across the propofol dosage.

Nominal logistic regression: propofol concentrations versus BIS Scores

In table 1 nominal logistic regression analysis has been applied to model the relationship between propofol concentrations and BIS values. Positive coefficient value for logit 1 indicates that as BIS score increases propofol concentration tends to be in propofol concentration 1.2 µg/mL compared to propofol concentration 1.6 µg/mL. Since all coefficients are positive, the conclusions are all same. p-value = 0.000 for all coefficients show that BIS score affects being in propofol concentrations 1.2, 0.8, 0.4 and 0 µg/mL compared to concentration 1.6 µg/mL. The results reveal that the odds increase when we compare lower concentrations with concentration 1.6 µg/mL. Nominal logistic regression has been chosen over ordinal logistic regression since effect of BIS are not same for each concentration of propofol which is obvious from the larger regression coefficients and odds ratios for logit 2, 3 and 4.

**Table 1:** Logistic regression table with coefficients and confidence intervals for each propofol concentrations*.

						95% CI	
						
Predictor	Coef	SE Coef	Z	P	Odds Ratio	Lower	Upper
Logit 1: (1.2µg/mL / 1.6 µg/mL) Constant BIS scores	-1.892 0.021	0.167 0.002	-11.30 9.86	0.000 0.000	1.02	1.02	1.03

Logit 2: (0.8 µg/mL / 1.6 µg/mL) Constant BIS scores	-7.875 0.098	0.191 0.002	-41.24 41.98	0.000 0.000	1.10	1.10	1.11

Logit 2: (0.4 µg/mL / 1.6 µg/mL) Constant BIS scores	-18.485 0.215	0.289 0.003	-64.04 65.06	0.000 0.000	1.24	1.23	1.25

Logit 2: (0 µg/mL / 1.6 µg/mL) Constant BIS scores	-20.329 0.226	0.397 0.004	-51.15 51.04	0.000 0.000	1.25	1.24	1.26

*Propofol dosage 1.6 µg/mL* has been chosen as the reference level. The Coef. (coefficient) column shows the change in the logit of the given dosage compared to dosage 1.6* µg/mL* when BIS values increse by 1 unit. BIS, bispectral index; CI, confidence interval; SE, standard error.*

**Figure 3:**
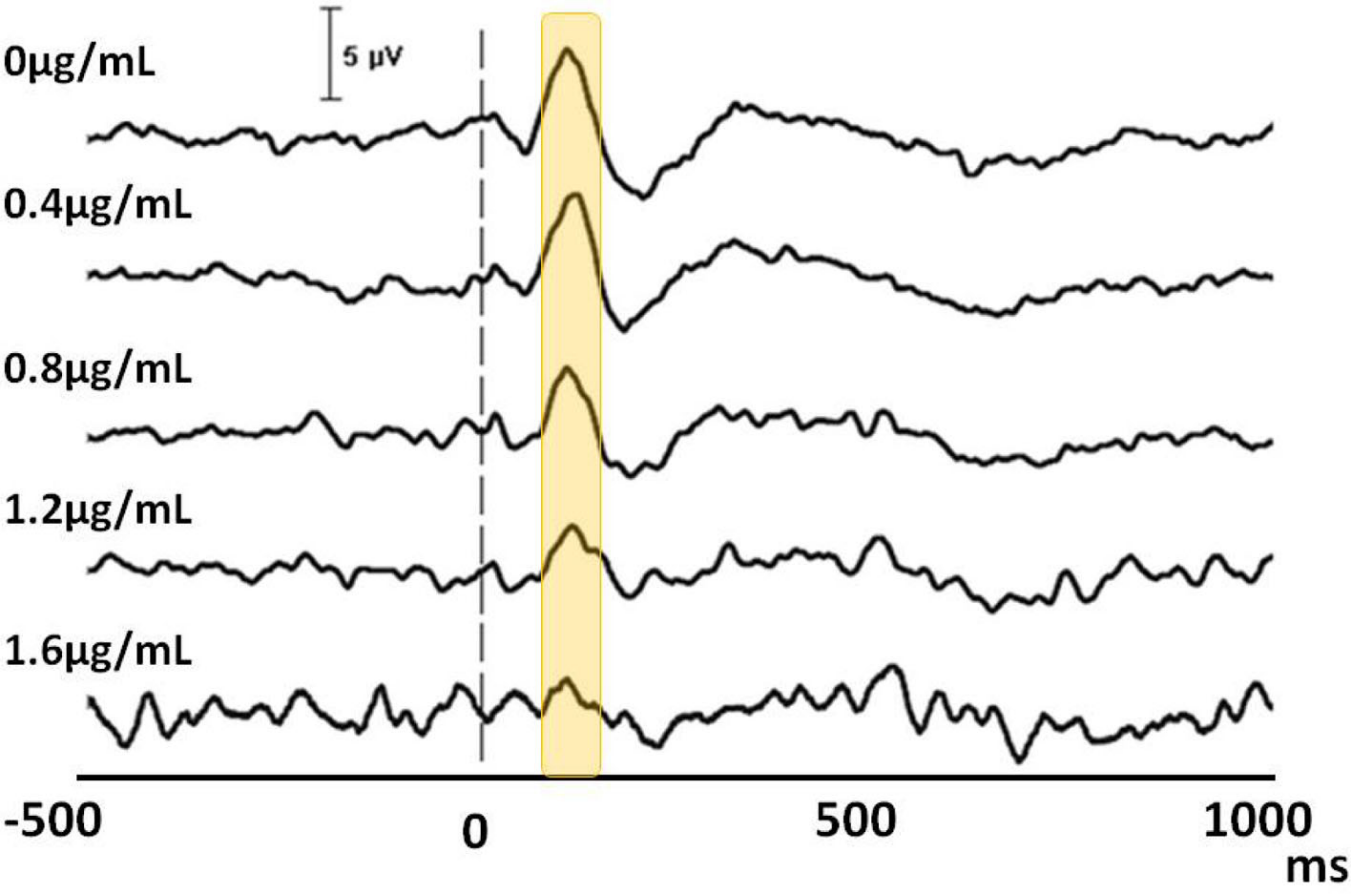
**The auditory N1 component in sleep.** The waveforms represent grand average (N=12) of electrophysiological recordings during anesthesia. The horizontal axis denotes the temporal domain from prestimulus 500 ms to poststimulus 1000 ms. The 0 marks the point of auditory stimulation.

Accordingly, the increments of propofol resulted in decreasing of the BIS index (p<0.0001) (Table 2). The auditory stimulations were presented throughout the session. The evoked waveforms are presented in figure 3. Here, the upper waveforms represent the averaged response with a distinct N1 component (yellow vertical box points out). The lower lines represent the increasing level of propofol. Accordingly, the highest level of propofol displays no clear N1 waveform. In order to highlight the differences between the conditions, the lower dosages (0.4 and 0.8 µg/mL) and the higher dosage levels (1.2 and 1.6 µg/mL) were grouped. The comparison of higher dosage group with the lower one revealed a significant difference (p=0.003).

**Table 2:** The N1 amplitudes corresponding to different propofol target concentrations (SE are given within the brackets).

Propofol	N1 (µV)
**0.0 µg/mL**	-2.24 (0.67)
**0.4 µg/mL**	-1.98 (0.61)
**0.8 µg/mL**	-1.40 (0.37)
**1.2 µg/mL**	-1.04 (0.66)
**1.6 µg/mL**	-0.39 (0.38)

**Figure 4:**
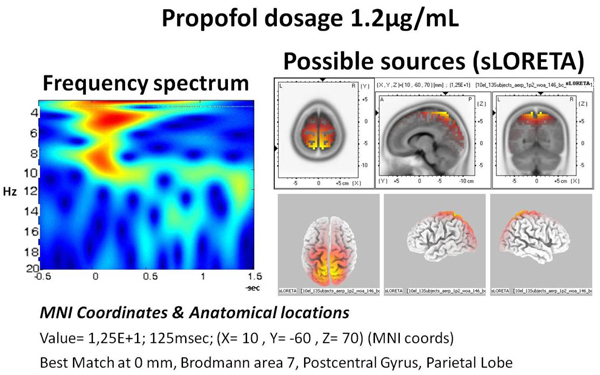
**The wavelet spectrogram and the sLORETA sources of anesthesia.** The spectrogram (left) has seconds in horizontal axis, whereas the 0 value represent the point of auditory stimulation. The vertical axis denotes the frequency increasing towards bottom (Hz). The possible sources are presented at right side with a sLORETA power maxima matching the 100 ms (N1).

**Figure 5:**
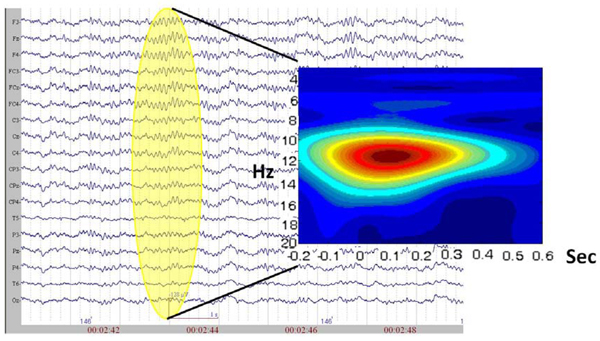
**The EEG recording and the spectrogram of the spindle oscillations.** The EEG was obtained during a propofol level around 0.8 µg/mL, The spectrogram shows a spindle oscillation at around 11-14 Hz which lasts slightly under 1 sec.

A sample sLORETA of group average is provided in figure 4. On the left hand side, the spectrogram of the evoked response for N1 is given. The 0 denotes the stimulus onset time. The major part of the activity lies from delta to alpha ranges (3.5-11 Hz). The sLORETA reveals highest power to be located at Broadman Area (BA) 6. Additionally, with the administration of propofol, especially reaching around 0.8 µg/mL, the distinctive spindle oscillations at around 11-14 Hz were observed. These waveforms lasted approximately for 1 second and were predominantly present in the central line electrodes (Figure 5).

Lower propofol dosage (0.4 and 0.8 µg/mL) level revealed sLORETA locations of maximal activity areas to include, BA6 (5 subjects), BA7 (7 subjects), BA20 (3 subjects), BA3 (1 subject), BA11 (1 subjects), BA18 (3 subject), BA19 (1 subject), BA38 (1 subject), BA47 (2 subjects).

For the higher propofol dosage (1.2 and 1.6 µg/mL) the locations from the maximal activity areas included, BA6 (6 subjects), BA7 (7 subjects), BA3 (1 subject), BA8 (2 subjects), BA11 (2 subjects), BA19 (2 subjects), BA20 (1 subject), BA21 (1 subject), BA22 (1 subject), BA37 (1 subject).

### The sleep experiment

**Figure 6:**
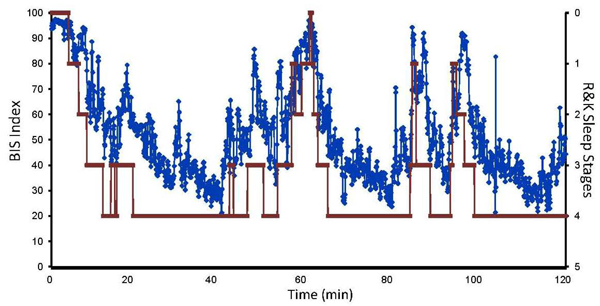
**The comparison of BIS and R&K sleep stages.** The right vertical axis represents the BIS values (0 to 100). The horizontal axis represent the temporal values (minutes). The secondary vertical axis represents the sleep stages. The thick lines (red) denote the R&K stage and the blue dots represent the BIS values.

The Bispectral Index (BIS) measurements have been utilized both for anesthesia and sleep conditions. The BIS values follow the depth of sleep closely. The figure 6 presents the BIS (blue) and classical R&K stages (red) in vertical axes.

According to Spearman analysis, the correlation rate between BIS values and sleep stages in this sleep period was r =0.82.

### The microstates of sleep and possible effect of stimulations

**Figure 7:**
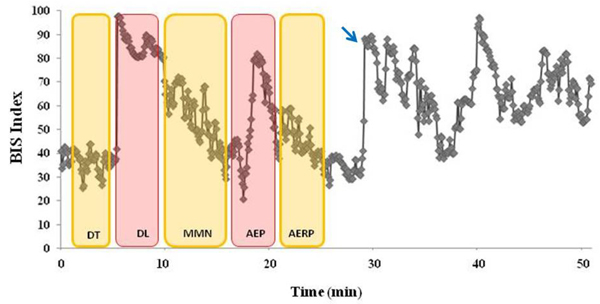
**The brain biophysics battery application during sleep monitored via BIS.** The raw BIS index during sleep (gray dots) is shown in line. The vertical bars represent the time points where the auditory blocks were presented (DT: dichotic tone; DL: dichotic listening; MMN: mismatch negativity; AEP: auditory evoked potentials; AERP: auditory event related potential). The arrow points to a sudden increase of BIS values upon application of dichotic syllables.

During the administration of auditory stimulations, upon changing of the type of stimulation of BBB, we have observed changes in depth of sleep of subjects. These changes have been noted e.g. figure 7 especially with the presentation of syllables (DL) after simple tones (DT). The second application of syllables resulted in a similar increase in BIS values (arrow in Fig. 7). As every subject would have a unique sleep pattern-and it is not predictable- the causal relationship of external stimulation changes on the sleep patterns remains a further study topic.

### Auditory stimulations during sleep

**Figure 8:**
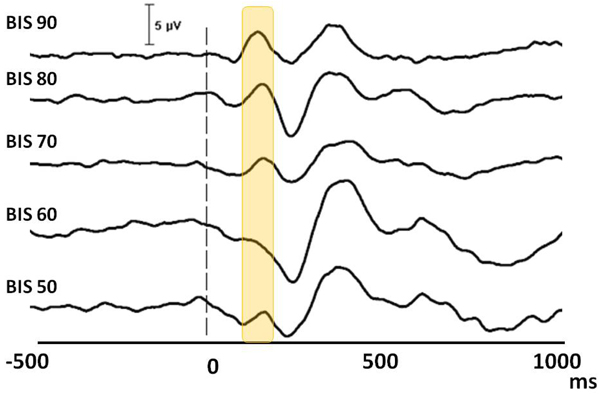
**The auditory N1 component in sleep.** The waveforms represent grand average (N=12) of electrophysiological recordings. The horizontal axis denotes the temporal domain from prestimulus 500 ms to poststimulus 1000 ms. The 0 marks the point of auditory stimulation. The yellow bar represents the N1 waveform.

The auditory evoked potentials have been succesfully obtained during different stages of sleep. The figure 8 and Table 3 represent the N1 potential across different BIS values. “The lower the BIS value deeper the sleep” was already obtained above (figure 6) [26]. Therefore the lower BIS values in figure 8 represent deeper sleep stages through which the N1 wave starts to dissappear.

**Table 3:** The N1 amplitudes corresponding to different sleep depth (via BIS index) (SE are given within the brackets).

BIS	N1 (µV)
**90**	-0.9 (1.04)
**80**	-0.6 (0.42)
**70**	0.6 (0.70)
**60**	1.1 (0.62)
**50**	1.4 (0.71)

Similar to the anesthesia, the higher BIS group (80 and 70) and the lower BIS group (60 and 50) were grouped. The comparison of higher BIS group with the lower one revealed a significant difference (p=0.005).

**Figure 9:**
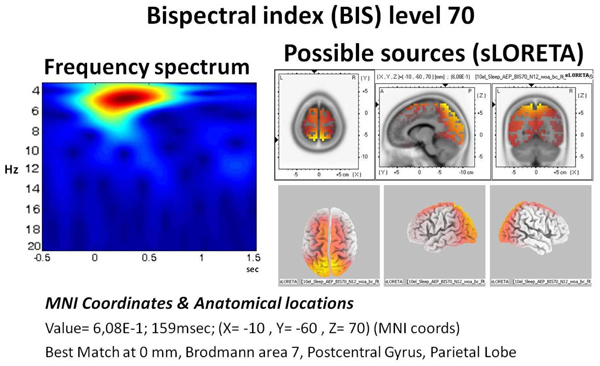
**The wavelet spectrogram and the sLORETA sources of sleep.** The spectrogram (left) has seconds in horizontal axis, whereas the 0 value represent the point of auditory stimulation. The vertical axis denotes the frequency increasing towards bottom (Hz). The possible sources are presented at right side with a sLORETA power maxima matching the 100 ms (N1).

A sample sLORETA of group average is provided in figure 9. On the left hand side, the spectrogram of the evoked response for N1 during sleep is given. The major part of the activity lies around delta with some activity towards theta ranges (3.5-7 Hz). The sLORETA reveals highest power to be located at Broadman Area 7.

## Discussion

The current manuscript presents data from different experimental setups and from different conscious states. Therefore a confined approach was applied. Accordingly, the effect of simple auditory stimulations in the range of 100-150 ms was focused. The anesthesia procedure by means of step-wise administration of propofol, has displayed a gradual loss of N1. Likewise with deepening of sleep -which could be monitored also with BIS- the N1 component diminished.

It has been reported that approximately after the 100-150 ms of the auditory stimulation a waveform which has a negative peak was observed in awake subjects [6,27]. This waveform is called (N100) “N1”. The neuronal generators for this waveform are suggested to be *planum temporale* in the secondary cortical area [28].

The current results revealed activities located at around BA3, BA6, BA7, BA8, BA11, BA19, BA20, BA21, BA22, BA37, BA38 and BA47 regions during the auditory stimulations under propofol anesthesia. These locations were found across the subjects using the GFP maxima matching the N1 peak (113 ms). The presence of BA6 and BA7 were more frequent than the other locations. The BA7 area had been located in other studies highlighting the functional prospects of precuneus [29-31]. Additionally, the vegetative state has been linked to impaired activity at this area using PET imaging [32].

Furthermore the current sleep experiment findings revealed locations around BA4, BA7, and BA9. Likewise, in the study of Anderer [22], the sleep spindles which are more frequent in Stage 2 (and thus around BIS level 70) were found to be related to BA9 and BA10. In [22], BA7, BA9, and BA10 were among the common activation areas. The activation of these areas has been linked to sleep spindles. Accordingly the areas from the current study are plausible target areas of sleep and anaesthesia domains. Interestingly, the sleep and anaesthesia share similar areas to a degree for the functional changes. The short term spectral changes like in the case of spindles, need to be further addressed in parallel experimental designs.

With the administration of propofol, especially reaching around 0.8 µg/mL the distinctive spindle oscillations at around 11-14 Hz were observed. At this level also the subjects started losing their consciousness and secondly the N1 waveform began to disappear. While the formation of LOC, the disappearance of N1 and the appearance of spindle oscillations can be coincidental, there have been various reports supporting the case [33-35]. The presence of this type oscillatory activity has been attributed to inductive versus disruptive properties [35-38]. Therefore the dual nature of these activities needs to be unveiled via controlled studies.

The sleep related EEG segments and the spectrograms also revealed spindle activity at around BIS levels of 80-70. The causal mechanisms and relationship of these spindles to anesthesia are beyond the scope of current manuscript, however there have been various studies indicating functional aspects of the spindles [22,33,35,39,40].

The spatiotemporal properties of brain functioning undergo some degree of changes during alterations in the conscious states. Some of these mechanisms may be linked to minor to large scale networks in the brain [41-43]. These changes may be necessary for brain to shift from one state to another (i.e. sleep), or they can be direct effects of medications (i.e. propofol anesthesia).

### Does auditory cognitive processing cessate in deep anesthesia?

The disappearance of basic electrophysiological patterns does not necessarily point to cessation of auditory cognitive processing. This could be analyzed with implicit memory paradigms as well as other special designs. Henceforth, caution is necessary while commenting on electrophysiological data in relationship to cognition.

### Sleep dynamics

The first stage results have provided a basic understanding in these altered states such that various auditory stimuli are successfully processed in both light and deep sleep stages. In addition to information processing during sleep, a large group of researchers is concerned with sleep disorders [44-46]. However; all these studies are still far away from elucidating the sleep field. In order to shed light on sleep processes, the brain functioning in wakefulness shouldn’t be completely neglected. It has been indicated that sleep has a marked effect on N1 waveform [28]. Researchers have stated that at the beginning of sleep there is a prolongation in latency and decrease in amplitude of N100 component [29,47]. It has been also reported that N100 waveform is highly sensitive to physical properties of the stimulus and with the repetition of the same stimuli N100 amplitude decreases [48]. It was stated that during NREM sleep amplitude of N100 decreases and the latency lengthens [49]. In a research it has been found that the amplitude of N100 reduces in slow wave sleep and slightly increases in REM sleep [50]. Näätänen has pronounced that in alert wakefulness the N100 component is correlated with task performance [51]. As can be seen from the above mentioned studies, many researchers have pointed that N100 waveform is susceptible to the state of the brain. A common feature of stage 2, the sleep spindles, is thought to be related with suppression of information processing [52]. It has been associated that decrease in N100 amplitude in N2 sleep to the aforementioned function of the sleep spindles [53]. Atienza et al. have interpreted the decrease of N100 amplitude as reduced afferent sensorial processing at the subcortical level [47] In the light of these results one could explain the decrease in N100 amplitude in deeper sleep stages in our study to be due to the suppression of auditory stimulation in order to maintain sleeping.

From a higher scope, the stages of sleep are artificial separations, whereas during natural sleep the brain may shift abruptly from one state to another. The external and internal conditions may all play a role in this sleep equilibrium. These shorter term stages may be defined as microstates. The external stimulations do have effect on immediate neurocognitive properties. Additionally, these stimulations may also play a causative role for the brain change its response state. Therefore, the observations that we have stated in our study will remain as a study area for our group. The brain biophysics battery is made up of different auditory stimulation blocks. Hence the different properties of brain responsiveness can be addressed while the block changes might serve to highlight the microstates level.

### Future research agenda

The field of studying brain functioning as in the present approach, would benefit from further experiments or research methods. These can be enriched by incorporating “Directed information transfer”, “Entropy and coherence”, “Causal relationships”, and “Brain asymmetry (i.e. dichotomy) under different conditions”. Additionally, an extended inclusion of various parameters such as body temperature and other physiological data might be beneficial to explain further mechanisms of the brain that interact with the body.

## Conclusions

Both sleep and anesthesia are dynamic conditions which require a uniform approach. Auditory stimulations with distinct features may provide a thorough insight into the brain responsiveness in different conscious states. Additionally spectro-temporal properties of the dynamic states and the analysis of microstates can constitute to better understanding of underlying mechanisms. The other line of plausible applications includes the neuropsychiatric pathologies, coma, and other major states of the brain.

## Competing interests

The Embedded Interactive Stimulus Unit (EMISU) system has a “patent pending” status.

## Authors' contributions

MO conceived of the study, and constructed its design and coordination. OB participated in the anesthesia experiments and supported the sLORETA assessments, SK participated in the sleep experiments and supported with the analysis of the data, NG participated in the anesthesia experiments and AO participated in the design of the study and manuscript. All authors read and approved the final manuscript.

## List of abbreviations

ABR: Auditory Brain-stem Responses; AEP: Auditory Evoked Potentials; AERP: Auditory Event Related Potential; BA: Broadman Area; BBB: Brain Biophysics Battery; BIS: Bispectral Index; CDV: Current Density Value; DL: Dichotic Listening; DT: Dichotic Tone; ECoG: Electrocorticogram; EEG: Electroencephalography; EMISU: Embedded Microcontroller Interactive Stimulus Unit; EOG: Electrooculography; fMRI: functional Magnetic Resonance Imaging; GFP: Global Field Power; ISI: Inter Stimulus Interval; LOC: Loss Of Consciousness; MEG: Magneto Encephalography; MLRs: Middle Latency Responses; MNI: Montreal Neurological Institute; MMN: Mismatch Negativity; PET: Positron Emission Tomography; R&K: Rechtschaffen and Kales; ROI: Region Of Interest; sLORETA: standardized Low Resolution Electromagnetic Tomography; SPL: Sound Pressure Level; SQI: Signal Quality Index)

## References

[B1] Wang Y Liu D Discovering the capacity of human memory. Brain and Mind 2003 4 2 189 198 10.1023/A:1025405628479

[B2] Plourde G Auditory evoked potentials. Best Pract Res Clin Anaesthesiol 2006 20 1 129 139 10.1016/j.bpa.2005.07.012 16634420

[B3] Jones JG Konieczko K Hearing and memory in anaesthetised patients. Br Med J (Clin Res Ed) 1986 292 1291 1293 10.1136/bmj.292.6531.1291 3085820 PMC1340306

[B4] Näätänen R The mismatch negativity: a powerful tool for cognitive neuroscience. Ear Hear 1995 16 1 6 18 7774770

[B5] Fudickar A Kluzik A Weiler N Scholz J Tonner PH Bein B A comparison of auditory evoked potentials derived from a monitor integrated module versus standard technique. J Neurosurg Anesthesiol 2009 21 2 120 126 10.1097/ANA.0b013e3181990d00 19295390

[B6] Bayazit O Oniz A Hahn C Güntürkün O Ozgoren M Dichotic listening revisited: Trial-by-trial ERP analyses reveal intra- and interhemispheric differences. Neuropsychologia 2009 47 536 545 10.1016/j.neuropsychologia.2008.10.002 18955072

[B7] Hugdahl K Symmetry and asymmetry in the human brain. Eur Rev 2005 3 2 119 133 10.1017/S1062798705000700

[B8] Hugdahl K Hugdahl K. & Davidson RJ Dichotic listening in the study of auditory laterality. The Asymmetrical Brain 2005 MIT Press 441 476

[B9] Ozgoren M Recording neuroelectrical activity under hostile surgical environment Int J Psychophysiol 2008 69 3 160 10.1016/j.ijpsycho.2008.05.407

[B10] Gokmen N Oniz A Bayazit O Erdogan U Akkan T Ozkurt A Ozgoren M The assessment of acoustical and electromagnetic noise on EEG monitoring during spinal surgery operations. J Neurol Sci Turk 2009 26 4 472 83 http://jns.dergisi.org/text.php3?id=322

[B11] Nasibov E Ozgoren M Ulutagay G Oniz A Kocaaslan S On the analysis of BIS stage epochs via fuzzy clustering. Biomed Tech (Berl) in press 20156029 10.1515/BMT.2010.009

[B12] Schneider G Hollweck R Ningler M Stockmanns G Kochs EF Detection of consciousness by electroencephalogram and auditory evoked potentials. Anesthesiology 2005 103 934 943 10.1097/00000542-200511000-00006 16249666

[B13] McNeer RR Bohόrquez J Ozdamar O Influence of auditory stimulation rates on evoked potentials during general anesthesia. Anesthesiology 2009 110 1026 1035 10.1097/ALN.0b013e31819dad6f 19352165

[B14] Nakamura M Uchida S Maehara T Kawai K Hirai N Nakabayashi T Arakaki H Okubo Y Nishikawa T Shimizu H Sleep spindles in human prefrontal cortex: an electrocorticographic study. Neurosci Res 2003 45 419 427 10.1016/S0168-0102(03)00007-5 12657455

[B15] Manshanden I De Munck JC Simon NR Lopes da Silva Fernando H Source localization of MEG sleep spindles and the relation to sources of alpha band rhythms. Clin Neurophysiol 2002 113 1937 1947 10.1016/S1388-2457(02)00304-8 12464331

[B16] Simpson TP Manara AR Kane NM Barton RL Rowlands CA Butler SR Effect of propofol anaesthesia on the event-related potential mismatch negativity and the auditory-evoked potential N1. Br J Anaesth 2002 89 3 382 388 10.1093/bja/aef175 12402715

[B17] Tung A Mendelson WB Anesthesia and sleep. Sleep Med Rev 2004 8 213 225 10.1016/j.smrv.2004.01.003 15144963

[B18] Rechtschaffen A Kales A A manual of standardized terminology, techniques, and scoring system for sleep stages of human subjects. Brain information service/Brain Research Institute 1968 University of California

[B19] Pakarinen S Takegata R Rinne T Huotilainen M Näätänen R Measurement of extensive auditory discrimination profiles using the mismatch negativity (MMN) of the auditory event-related potential (ERP). Clin Neurophysiol 2007 118 1 177 185 10.1016/j.clinph.2006.09.001 17070103

[B20] Ozgoren M Erdogan U Bayazit O Taslica S Oniz A Brain asymmetry measurement using EMISU (embedded interactive stimulation unit) in applied brain biophysics. Comput Biol Med 2009 39 10 879 88 10.1016/j.compbiomed.2009.07.001 19665111

[B21] Anderer P Gruber G Saletu B Klösch G Zeitlhofer J Pascual-Marqui c RD Non-invasive electrophysiological neuroimaging of sleep Int Congr Ser 2002 1232 795 800 10.1016/S0531-5131(01)00687-2

[B22] Anderer P Klösch G Gruber G Trenker E Pascual-Marqui RD Zeitlhofer J Barbanoj MJ Rappelsberger P Saletu B Low-resolution brain electromagnetic tomography revealed simultaneously active frontal and parietal sleep spindle sources in the human cortex. Neuroscience 2001 103 581 592 10.1016/S0306-4522(01)00028-8 11274780

[B23] Pascual-Marqui RD Michel CM Lehmann D Low resolution electromagnetic tomography: A new, method for localizing electrical activity in the brain. Int J Psychophysiol 1994 18 49 65 10.1016/0167-8760(84)90014-X 7876038

[B24] Pascual-Marqui RD Standardized low resolution brain electromagnetic tomography (sLORETA): Technical details. Methods Find Exp Clin Pharmacol 2002 24 Suppl D 5 12 12575463

[B25] Cek E Ozgoren M Savaci A Continuous time wavelet entropy of auditory evoked potentials. Comput Biol Meddoi:10.1016/j.compbiomed.2009.11.005 20022318 10.1016/j.compbiomed.2009.11.005

[B26] Ozgoren M Kocaaslan S Oniz A Analysis of non-REM sleep staging with electroencephalography bispectral index. Sleep Biol Rhythms 2008 6 249 255 10.1111/j.1479-8425.2008.00372.x

[B27] Oniz A Güdücü C Aydin B Ozgoren M Event-related delta and theta responses by tactile stimuli. J Neurol Sci Turk 2008 25 2 117 127 http:// http://jns.dergisi.org/text.php3?id=221

[B28] Bastuji H Garcia-Larrea L Evoked potentials as a tool for the investigation of human sleep Sleep Med Rev 1999 3 23 45 10.1016/S1087-0792(99)90012-6 15310488

[B29] Ozgoren M Bayazit O Gokmen N Oniz A Spectral pattern analysis of propofol induced spindle oscillations in the presence of auditory stimulations. Open Neuroimag Jsubmitted 10.2174/1874440001004010121 PMC3141347 21792383

[B30] Vogt BA Laureys S Posterior cingulate, precuneal & retrosplenial cortices: cytology & components of the neural network correlates of consciousness. Prog Brain Res 2005 150 205 217 full_text 16186025 10.1016/S0079-6123(05)50015-3 PMC2679949

[B31] Cavanna AE Trimble MR The precuneus: a review of its functional anatomy and behavioural correlates. Brain 2006 129 564 583 10.1093/brain/awl004 16399806

[B32] Laureys S Goldman S Phillips C van Bogaert P Aerts J Luxen A Franck G Maquet P Impaired effective cortical connectivity in vegetative state: preliminary investigation using pet. NeuroImage 1999 9 377 382 10.1006/nimg.1998.0414 10191166

[B33] Ferenets R Lipping T Suominen P Turunen J Puumala P Jäntti V Himanen SL Huotari AM Comparison of the properties of EEG spindles in sleep and propofol anesthesia. Proceedings of the 28th IEEE EMBS Annual International Conference 2006 10.1109/IEMBS.2006.259909 17945960

[B34] Lee U Mashour GA Kim S Noh G-J Choi BM Propofol induction reduces the capacity for neural information integration: Implications for the mechanism of consciousness and general anesthesia. Conscious Cogn 2009 18 56 64 10.1016/j.concog.2008.10.005 19054696

[B35] Mackenzie L Pope KJ Willoughby JO Pysiological and pathological spindling phenomena have similar regional EEG power distributions. Brain Res 2004 1008 92 106 10.1016/j.brainres.2004.01.084 15081386

[B36] Palva S Palva JM New vistas for α-frequency band oscillations Trends Neurosci 2007 30 150 158 10.1016/j.tins.2007.02.001 17307258

[B37] Klimesch W Sauseng P Hanslmayr S EEG alpha oscillations: the inhibition–timing hypothesis Brain Res Rev 2007 53 63 88 10.1016/j.brainresrev.2006.06.003 16887192

[B38] Sherman SM Tonic and burst firing: dual modes of thalamocortical relay. Trends Neurosci 2001 24 122 126 10.1016/S0166-2236(00)01714-8 11164943

[B39] Huotari AM Koskinen M Suominen K Alahuhta S Remes R Hartikainen KM Jäntti V Evoked EEG patterns during burst suppression with propofol Br J Anaesth 2004 92 18 24 10.1093/bja/aeh022 14665548

[B40] Schabus M Hoedlmoser K Pecherstorfer T Anderer P Gruberb G Parapatics S Sauter C Kloesch G Klimesch W Saletu B Zeitlhofer J Interindividual sleep spindle differences and their relation to learning-related enhancements. Brain Res 2008 1191 127 135 10.1016/j.brainres.2007.10.106 18164280 PMC2855382

[B41] Bressler SL Tognoli E Operational principles of neurocognitive networks. Int J Psychophysiol 2006 60 139 148 10.1016/j.ijpsycho.2005.12.008 16490271

[B42] Fingelkurts AA Fingelkurts AA Kivisaari R Autti T Borisov S Puuskari V Jokela O Kähkönen S Increased local and decreased remote functional connectivity at EEG alpha and beta frequency bands in opioid-dependent patiens. Psychopharmacology 2006 188 42 52 10.1007/s00213-006-0474-4 16850117

[B43] Fingelkurts AA Kähkönen S Fingelkurts AA Kivisaari R Autti T Borisov S Puuskari V Jokela O Autti T Reorganization of the brain oscillations and their temporal characteristics during opioid withdrawal. J Psychopharmacol 2008 22 3 270 284 10.1177/0269881108089810 18541625

[B44] Baklan B Narcolepsy-cataplexy. J Int Med Sci 2007 3 26 19 26

[B45] Degirmenci E Bir LS Oncel C Unilateral thalamodiencephalic syndrome presenting with severe hypersomnia. J Neurol Sci Turk 2008 25 3 196 199 http://jns.dergisi.org/text.php3?id=230

[B46] Alemdar M Iseri P Kamacı S Efendi H Budak F Komsuoglu SS Risk factors and seasonal-diurinal variatons in lacunar infarctions. J Neuro. Sci Turk 2006 23 2 124 128 http://jns.dergisi.org/text.php3?id=95

[B47] Atienza M Cantero JL Escera C Auditory information processing during human sleep as revealed by event related brain potentials. Clin Neurophysiol 2001 112 2031 2045 10.1016/S1388-2457(01)00650-2 11682341

[B48] Loveless NE Brunia CMH Effects of rise-time on late components of the auditory evoked potential. J Psychophysiol 1990 4 369 380

[B49] Paavilainen P Cammann R Alho K Reinikainen K Sams M Näätänen R Johnson R, Rohrbaugh JW, Parasuraman R. Event related potentials to pitch change in an auditory stimulus sequence during sleep. Current trends in event-related potential research 1987 246 255 3480130

[B50] Bastuji H Garcia-Larrea L Franc C Mauguiere F Brain processing of stimulus deviance during slow-wave and paradoxical sleep: A study of human auditory evoked responses using the oddball paradigm. J Clin Neurophysiol 1995 12 155 167 7797630 10.1097/00004691-199503000-00006

[B51] Näätänen R The role of attention in auditory information processing as revealed by event-related potentials and other brain measures of cognitive function Behav Brain Sci 1990 13 201 208

[B52] Steriade M McCormick DA Sejnowski TJ Thalamocortical oscillations in the sleeping and aroused brain. Science 1993 262 679 685 10.1126/science.8235588 8235588

[B53] Elton M Winter O Heslenfeld D Loewy D Campbell K Kok A Event related potentials to tones in the absence and presence of sleep spindles. J Sleep Res 1997 6 78 83 9377537 10.1046/j.1365-2869.1997.00033.x

